# Metabolic network model guided engineering ethylmalonyl-CoA pathway to improve ascomycin production in *Streptomyces hygroscopicus* var. *ascomyceticus*

**DOI:** 10.1186/s12934-017-0787-5

**Published:** 2017-10-03

**Authors:** Junhua Wang, Cheng Wang, Kejing Song, Jianping Wen

**Affiliations:** 1 Key Laboratory of System Bioengineering (Tianjin University), Ministry of Education, Tianjin, 300072 People’s Republic of China; 2 SynBio Research Platform, Collaborative Innovation Center of Chemical Science and Engineering (Tianjin), School of Chemical Engineering and Technology, Tianjin University, Tianjin, 300072 People’s Republic of China

**Keywords:** Ascomycin, FK520, Metabolic network model, Ethylmalonyl-CoA pathway, *Streptomyces hygroscopicus* var. *ascomyceticus*

## Abstract

**Background:**

Ascomycin is a 23-membered polyketide macrolide with high immunosuppressant and antifungal activity. As the lower production in bio-fermentation, global metabolic analysis is required to further explore its biosynthetic network and determine the key limiting steps for rationally engineering. To achieve this goal, an engineering approach guided by a metabolic network model was implemented to better understand ascomycin biosynthesis and improve its production.

**Results:**

The metabolic conservation of *Streptomyces* species was first investigated by comparing the metabolic enzymes of *Streptomyces coelicolor* A3(2) with those of 31 *Streptomyces* strains, the results showed that more than 72% of the examined proteins had high sequence similarity with counterparts in every surveyed strain. And it was found that metabolic reactions are more highly conserved than the enzymes themselves because of its lower diversity of metabolic functions than that of genes. The main source of the observed metabolic differences was from the diversity of secondary metabolism. According to the high conservation of primary metabolic reactions in *Streptomyces* species, the metabolic network model of *Streptomyces hygroscopicus* var. *ascomyceticus* was constructed based on the latest reported metabolic model of *S. coelicolor* A3(2) and validated experimentally. By coupling with flux balance analysis and using minimization of metabolic adjustment algorithm, potential targets for ascomycin overproduction were predicted. Since several of the preferred targets were highly associated with ethylmalonyl-CoA biosynthesis, two target genes *hcd* (encoding 3-hydroxybutyryl-CoA dehydrogenase) and *ccr* (encoding crotonyl-CoA carboxylase/reductase) were selected for overexpression in *S. hygroscopicus* var. *ascomyceticus* FS35. Both the mutants HA-Hcd and HA-Ccr showed higher ascomycin titer, which was consistent with the model predictions. Furthermore, the combined effects of the two genes were evaluated and the strain HA-Hcd-Ccr with *hcd* and *ccr* overexpression exhibited the highest ascomycin production (up to 438.95 mg/L), 1.43-folds improvement than that of the parent strain FS35 (305.56 mg/L).

**Conclusions:**

The successful constructing and experimental validation of the metabolic model of *S. hygroscopicus* var. *ascomyceticus* showed that the general metabolic network model of *Streptomyces* species could be used to analyze the intracellular metabolism and predict the potential key limiting steps for target metabolites overproduction. The corresponding overexpression strains of the two identified genes (*hcd* and *ccr*) using the constructed model all displayed higher ascomycin titer. The strategy for yield improvement developed here could also be extended to the improvement of other secondary metabolites in *Streptomyces* species.

**Electronic supplementary material:**

The online version of this article (doi:10.1186/s12934-017-0787-5) contains supplementary material, which is available to authorized users.

## Background

Ascomycin (FK520) is a 23-membered polyketide macrolide antibiotic with immunosuppressant activity, which has similar characterization to FK506 (tacrolimus), exhibiting various therapeutic applications, including antifungal [1], immunosuppressive [2], antimalarial [3], antispasmodics [3] and several other uses [4, 5]. Meanwhile, many of its derivatives also have important pharmacological value, such as pimecrolimus, which has been confirmed to be effective in the treatment of various inflammatory skin diseases [4, 6]. Due to its broad clinical applicability and promising pharmaceutical prospects, ascomycin and its derivatives have recently attracted much attention of many researchers.

Recently, considerable efforts have been made to enhance the ascomycin titer of the production strains, including random mutagenesis and selection [7], fermentation technology optimization [8], comparative metabolic profile analysis [9], and so on. However, the relatively low yield of ascomycin in bio-fermentations is still a major obstacle for its further industrialization and commercialization. This problem may be due to our poor understanding of the mechanism of ascomycin overproduction as well as a lack of methods to efficiently identify the key-limiting steps as targets for strain engineering. Recently, genome-scale metabolic network model (GSMM) [10] has been developed as an effective strategy to analyze the intracellular metabolic behavior and identify metabolic engineering targets for efficient strain improvement. It has been successfully applied to the design of metabolic engineering strategies for a number of valuable products, such as succinic acid [11], spinosad [12], tacrolimus [13], riboflavin [14], dicarboxylic acid [15], putrescine [16], and so on.

*Streptomyces coelicolor* A3(2), the model organism of the *Streptomyces* genus, is the first one to be completely sequenced species of the genus *Streptomyces* [17]. The GSMM of *S. coelicolor* A3(2) was first constructed in 2005 (iIB711) [18], and was updated in 2010 (iMA789) [19] and 2014 (iMK1208) [20]. During the construction of the model iIB711, the authors analyzed the universality of the model by comparing the genome of *S. coelicolor* A3(2) with that of *Streptomyces avermitilis* [18]. It was found that 78% ORFs in the model were synteny conserved with *S. avermitilis* and some of the non-conserved ORFs might have synteny-conserved isoenzymes with *S. avermitilis*. The results illustrated that metabolic genes were highly conservative in the above two *Streptomyces* strains. In addition, it has been demonstrated that the size of panmetabolism (the set of all reactions in the investigated strains) in *Escherichia coli* was limited to the set of reactions already known and approached a constant [21]. This phenomenon may be due to the conservation of metabolic genes in these organisms, as well as our limited knowledge of gene functions and protein biochemical characteristics [21, 22]. However, in *Streptomyces* species, the size of panmetabolism might not approach a plateau because they contain large number of genes in their secondary metabolism. Nevertheless, the primary metabolic genes in *Streptomyces* species are more conserved, which has been illustrated by comparing genome and pangenome analysis [23, 24]. Thus, one can expect that a newly sequenced *Streptomyces* strain would not significantly expand the size of the set of known primary metabolic reactions in *Streptomyces* species. That is, the construction of a general metabolic network model for *Streptomyces* strains only requires the addition of limited diversity to the current primary metabolic network of the model strain *S. coelicolor* A3(2).

Nevertheless, the metabolic conservation of *Streptomyces* species has so far not been fully analyzed, and the primary reactions in model iMK1208 of *S. coelicolor* A3(2) have not been investigated to establish if these reactions are conserved and could be applied to construct a general metabolic network model for *Streptomyces* strains. To resolve these issues, the metabolic enzymes in the updated model iMK1208 of *S. coelicolor* A3(2) were compared with those of 31 *Streptomyces* strains. And high conservation of the primary metabolic enzymes had been found in the all selected *Streptomyces* strains. Then, a metabolic network model of *S. hygroscopicus* var. *ascomyceticus* for ascomycin biosynthesis was attempted to construct by using the universal primary metabolic reactions from model iMK1208 and adding the reactions associated with the synthesis of ascomycin in *S. hygroscopicus* var. *ascomyceticus*. Using the thus constructed metabolic network model, some potential targets for ascomycin overproduction were predicted with the aid of FBA [25] and MOMA [26]. Among all the potential targets, two target genes (i.e. *hcd* and *ccr*) located in the ethylmalonyl-CoA pathway showed high promoting effects on ascomycin overproduction. After gene overexpression manipulation, all the engineered strains had shown higher ascomycin production than control, and the highest ascomycin titer reached to 438.95 mg/L in the strain HA-Hcd-Ccr. These findings not only suggested a general metabolic network model could be construed for *Streptomyces* species, but also provided an effective method for the titer improvement of other products in different *Streptomyces* species.

## Methods

### Conservation analysis of the proteins in iMK1208 against *Streptomyces* species

A total of 31 *Streptomyces* strains with completely sequenced genomes (deposited in the NCBI by September 2015) were used in the conservation analysis. All the available protein coding sequences of the 31 *Streptomyces* strains were retrieved from the National Center for Biotechnology Information (NCBI), and plasmids were omitted from the analysis. The accession numbers of all 31 strains are listed in Additional file 1. The protein sequences of *S. coelicolor* A3(2) in metabolic model iMK1208 were used as the reference, and the sequence similarities on protein level were calculated from a pair-wise comparison of homologous sequences between the protein sequences in the model iMK1208 and the protein coding sequences of the 31 *Streptomyces* strains using BLAST v2.2.30+. The top hit for each protein sequence (i.e. lowest e value) was selected as the optimal comparison result. The protein sequences were considered as conserved at the threshold of 40% identity, 50% of query protein length aligned and e value < 1e − 05.

### Metabolic network construction

The initial model of *S. hygroscopicus* var. *ascomyceticus* FS35 was constructed based on the model iMK1208 of *S. coelicolor* A3(2). Several secondary metabolism subsystems actinorhodin, undecylprodigiosin, calcium dependent antibiotic, and germicidin biosynthesis were removed from the model iMK1208. Some specific secondary metabolites biosynthesis reactions in other subsystems such as membrane transport, cofactor and prosthetic group biosynthesis were also removed from the model. The reactions related with ascomycin specific precursors biosynthesis and overall biosynthesis reactions were added to the new model.

As a kind of constraints-based metabolic model, the necessary constraints were used to obtain the optimal flux solution space. For example, 6-phosphogluconate dehydrogenase was shown to be NADP-dependent and no detectable activity with NAD [27], the upper bounds of reaction GND2 (NAD-dependent) and GND (NADP-dependent) were set to 0 and 1000, respectively. Based on the way for glucose entry and phosphorylation, the upper bound of reaction G1DH was set to 0 [27, 28]. According to the analysis of the carbon-flux distribution in *S. coelicolor* [27], *S. hygroscopicus* var. *ascomyceticus* [29] and *S. lividans* [30], the lower bound of reaction PGL (catalyzed by 6-phosphogluconolactonase) was set to 0.2, which means that the lowest flux through pentose phosphate pathway was 20% of the available glucose-6-phosphate. Additionally, the lower bound of the anaplerotic reaction PPC was set to 0.1 according to the carbon-flux distribution of *S. coelicolor* [27]. Menaquinone biosynthesis pathway was also further improved according the Kyoto Encyclopedia of Genes and Genomes (KEGG) database. The details of the all reactions and metabolites in the models are provided in Additional file 2.

### Identification of the overexpression targets

Flux balance analysis (FBA) was performed to calculate the flux distributions throughout the metabolic network by defining a maximized-biomass equation as the objective function using COBRAToolbox-2.0 [31] in MATLAB [32–35]. To further validate the model, the predicted specific growth rates in a glucose-limited minimal medium were compared with the observed growth in glucose limited environments [36]. The algorithm developed by Boghigian et al. was applied for the identification of overexpression targets [37]. First, FBA was used to calculate the initial flux distribution for the network by defining the maximized specific growth rate as the objective function. Subsequently, each non-zero reaction flux obtained by FBA was amplified to some extent, and MOMA was performed to search for the minimal Euclidean distance between the original model and the model after gene perturbation by solving a quadratic programming problem. The overexpression targets were identified by comparing the *f*_PH_ value (Eq. 1), defined as the ratio of the weighted and dimensionless specific growth rate and the specific ascomycin production rate. Overexpression targets with higher *f*_PH_ values were regarded as better candidates for experimental manipulation.

1 $$\begin{aligned} f_{\text{PH}} & = \left( {f_{\text{biomass}} } \right)\, \times \,\left( {f_{\text{ascomycin}} } \right) \\ & = \left( {\frac{{v_{{{\text{biomass}}, {\text{overexpression}}}} }}{{v_{{{\text{biomass}}, {\text{wild}}}} }}} \right)\left( {\frac{{v_{{{\text{ascomycin}}, {\text{overexpression}}}} }}{{v_{{{\text{ascomycin}}, {\text{wild}}}} }}} \right) \\ \end{aligned}$$

### Bacterial strains, plasmids, and cultural conditions

All strains and plasmids used in this study are summarized in Table 1. The parent strain *S. hygroscopicus* var. *ascomyceticus* FS35 was isolated after femtosecond laser irradiation [7]. All *S. hygroscopicus* var*. ascomyceticus* mutants used in this study were derived from FS35. *Escherichia coli* JM109 was used to propagate all plasmids. *E. coli* ET12567/pUZ8002 was used as the nonmethylating plasmid donor strain for intergeneric conjugation with FS35. All *E. coli* strains were grown in Luria–Bertani (LB) medium at 37 °C. The integrative *E. coli*-*Streptomyces* shuttle vector pIB139 containing the ermE* promoter (*P*_ermE*_) was used for gene overexpression in *S. hygroscopicus* var. *ascomyceticus* FS35. *S. hygroscopicus* var. *ascomyceticus* strains was grown at pH 7.0 and 28 °C, MS medium was used for the preparation of *Streptomyces* spores suspensions, seed cultures were prepared as described previously [38]. Synthetic medium was used to culture the FS35 strain for model validation, and it contained 40 g/L dextrin, 20 g/L glucose, 1.0 g/L NaCl, 2.0 g/L (NH4)_2_SO_4_, 1.0 g/L K_2_HPO_4_·3H_2_O, 2.0 g/L CaCO_3_, 1.0 g/L MgSO_4_·7H_2_O, 0.001 g/L FeSO_4_·7H_2_O, 0.001 g/L MnCl_2_·4H_2_O, 0.001 g/L ZnSO_4_·7H_2_O, MOPS 5.0 g/L, pH 7.0. The production medium contained 20 g/L soluble starch, 40 g/L dextrin, 5.0 g/L peptone, 5.0 g/L yeast powder, 6.0 g/L corn steep liquor, 12 mL/L soybean oil, 1.0 g/L MgSO_4_·7H_2_O, 0.5 g/L MnSO_4_·1H_2_O, 1.5 g/L (NH4)_2_SO_4_, 0.5 g/L K_2_HPO_4_·3H_2_O, and 2.0 g/L CaCO_3_. *Streptomyces coelicolor* A3(2) were cultured in YEME at 28 °C for the isolation of genome. For conjugation experiments, *S. hygroscopicus* var. *ascomyceticus* strains were performed according to the *Streptomyces* standard protocols [39]. The transformants of *S. hygroscopicus* var*. ascomyceticus* were selected on 2CMY contained starch 10 g/L, casein tryptone 2.0 g/L, NaCl 1.0 g/L, (NH_4_)_2_SO_4_ 2.0 g/L, K_2_HPO_4_ 1.0 g/L, CaCO_3_ 2.0 g/L, FeSO_4_·7H_2_O 0.001 g/L, MgCl_2_·6H_2_O 0.001 g/L, ZnSO_4_·7H_2_O 0.001 g/L, agar 20 g/L, pH 7.0. Agar plates by overlaying with an appropriate antibiotic or combination of antibiotics.

**Table 1:** Bacterial strains and plasmids used in this study

Strains/plasmids	Relevant features	Source/reference
Strains		
*S. hygroscopicus* var. *ascomyceticus* FS35	Mutant, improved ascomycin producer	[7]
FS-PIB	FS35 transformed with pIB139	This study
HA-Ccr	FS35 transformed with pIB/ccr	This study
HA-Hcd	FS35 transformed with pIB/hcd	This study
HA-Hcd-Ccr	FS35 transformed with pIB/hcd/ccr	This study
*E. coli* DH5α	Plasmid construction and general cloning	Takara
*E. coli* ET12567	pUZ8002, nonmethylating plasmid donor, Cm^R^, Kan^R^	[40]
Plasmids		
pUC18	*E. coli* general cloning vector, Amp^R^	[41]
pIB139	Integrative *E. coli*-*Streptomyces* shuttle plasmid containing *oriT*, *attP*, *int*, *aac(3)IV* and ermEp*	[42]
pIB/ccr	pIB139 based integrative plasmid containing *ccr* gene, Apr^R^	This study
pIB/hcd	pIB139 based integrative plasmid containing *hcd* gene, Apr^R^	This study
pUC/hcd	pUC18 based plasmid containing *hcd* gene	This study
pUC/hcd/ccr	pUC18 based plasmid containing *hcd* and *ccr* gene	This study
pIB/hcd/ccr	pIB139 based integrative plasmid containing *hcd*-*ccr* gene, Apr^R^	This study

### Cloning, plasmid construction and transformation

General DNA manipulation and intergeneric conjugation of *E. coli* with *Streptomyces* were performed according to standard protocols [39]. All the strains and primers used in this work are listed in Additional file 3: Table S1. A derivative of pSET152 [43], pIB139, was used for *hcd* and *ccr* gene overexpression. The plasmids pIB/ccr and pIB/hcd were constructed as follows. The coding sequence of *hcd* and *ccr* were amplified from the genomic DNA of *S. coelicolor* A3(2) by PCR using the primer pairs hcdF1/hcdR1 and ccrF1/ccrR1 respectively. The *hcd* and *ccr* PCR products were digested using the restriction enzymes *Nde*I and *Xba*I, and the resulting fragments produced by digestion were cloned into the pIB139 vector between *Nde*I and *Xba*I respectively to obtain pIB139R and pIB139D, respectively. The constructed plasmids pIB/hcd and pIB/ccr were both confirmed by DNA sequencing. The conformed plasmids pIB/hcd and pIB/ccr were introduced into the parent strain *S. hygroscopicus* var. *ascomyceticus* FS35 to obtain the *hcd* and *ccr* overexpression strains HA-Hcd and HA-Ccr, respectively. To construct the vector pIB/hcd/ccr, the primer pairs HcdF2 and HcdR2 were used for amplifying the gene *hcd* and the primer pairs CcrF2 and CcrR2 were used for amplifying the gene *ccr*. The *hcd* PCR product was digested using *Nde*I and *Pst*I, and then the fragment was cloned into the pUC18 vector to form pUC/hcd. The resulted plasmid pUC/hcd was cut using *Pst*I and *Xba*I, and linked with the *ccr* PCR product, which was digested with the same restriction enzymes, to form the plasmid pUC/hcd/ccr. The plasmid pUC/ccr/hcd was excised by *Nde*I/*Xba*I and the fragment including gene *hcd* and *ccr* was cloned into pIB139 to generate the plasmid pIB/hcd/ccr. The resulting plasmid was introduced into the parental strain *S. hygroscopicus* var. *ascomyceticus* FS35 via conjugal transfer, generating the overexpression strain HA-Hcd-Ccr. The parental strain *S. hygroscopicus* var. *ascomyceticus* FS35 with pIB139 (FS-PIB) was used as the negative control. All the positive mutants were confirmed by PCR amplification and DNA sequence analysis.

### Enzyme assays

The culture samples for enzyme activity analysis in vitro were harvested at the end of exponential phase (72 h) by centrifugation at 8000×*g* for 10 min at 4 °C. Cell pellets were washed 3 times by 100 mM Tris·HCl (pH 7.0) containing 5 mM MnSO_4_, 20 mM KCl, 2 mM DTT and 0.1 mM EDTA, and then resuspended in the same buffer [44]. Cell suspension was treated by ultrasonication for 10 min at 250 W for 5 cycles of 1 min each on ice bath. Supernatant obtained after centrifugation at 12,000×*g* for 20 min at 4 °C was stored at − 80 °C for assays of enzyme activity and total protein concentration. The concentration of total protein was detected by the Bradford method [45]. One unit (1 U) of enzyme activity was defined as the transformation of 1 μM/min.

3-Hydroxybutyryl-CoA dehydrogenase (Hcd, EC:1.1.1.157) activity was assayed by monitoring the decrease in NADH concentration at 340 nm using acetoacetyl-CoA as the substrate, the reaction mixture contained 100 mM Tris–HCl buffer (pH 7.5), 100 μM acetoacetyl-CoA, 150 μM NADH, and extract. The reaction was started by the addition of acetoacetyl-CoA [46, 47]. Activity of the crotonyl-CoA carboxylase/reductase (Ccr, EC:1.3.1.85) was assayed by the method reported previously [48].

### Analytical methods

Fermentations of *S. hygroscopicus* var. *ascomyceticus* FS35 and its derivatives were carried out as described previously [38]. Biomass concentration was determined by the measurement of dry cell weight (DCW). For the determination, 10 mL fermentation broth were sampled and then centrifuged at 8000×*g* for 10 min, washed once with 0.1 M HCl and twice with distilled water separately, and dried at 80 °C to constant weight. The concentration of ascomycin was quantitated by high-performance liquid chromatography (HPLC) analysis as described previously [38]. The residual total sugar in fermentation broth was quantified according to the phenol–sulfuric acid method using glucose as the standard [49].

## Results

### Metabolic conservation analysis in *Streptomyces* species

**Fig. 1:**
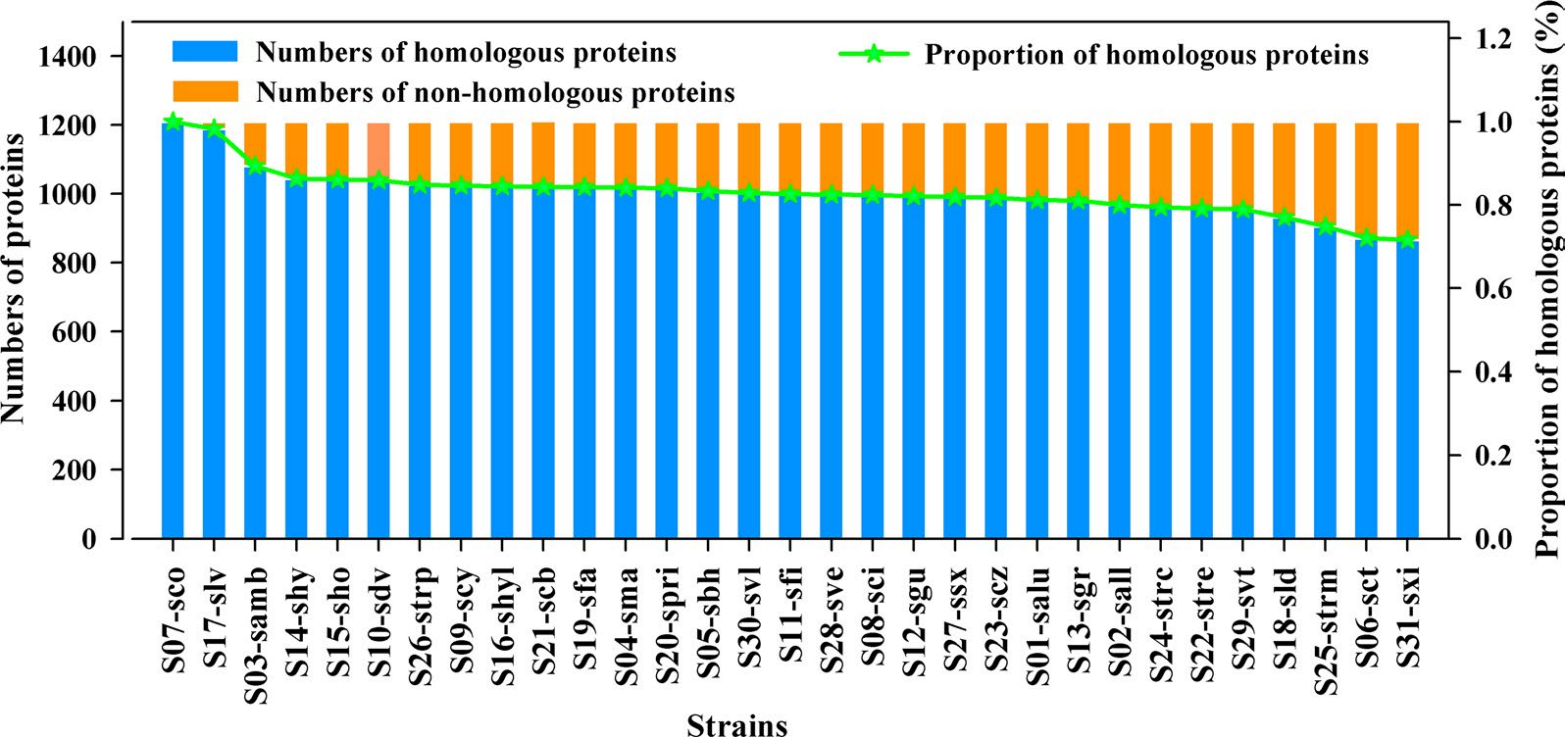
Conservation analysis of metabolic enzymes between *Streptomyces coelicolor* A3(2) and 31 *Streptomyces* strains. The proteins in the metabolic model iMK1208 of *S. coelicolor* A3(2) were used as a reference for protein conservation analysis of the 31 *Streptomyces* strains. The strains are indicated by the strain numbers in this study and their KEGG organism codes except for the strain *S. hygroscopicus limoneus* KCTC 1717, which is indicated by S16-shyl. S07-sco is short for *S. coelicolor* A3(2), which is the reference strain for conservation analysis

All the metabolic enzymes involved in the iMK1208 model of *S. coelicolor* A3(2), which is an important model strain for *Streptomyces* species, were used as the standard for metabolic conservation analysis of 31 *Streptomyces* strains. Except for the exchange and teichoic acid biosynthesis subsystems, the 1208 proteins in the other 43 metabolic subsystems of the model iMK1208 were preformed blastp homology analysis against 31 *Streptomyces* strains (Additional file 1). The names of these strains were constituted by the strain numbers in this study and the KEGG numbers except the strain *S. hygroscopicus limoneus* KCTC 1717. As shown in Fig. 1, 1208 metabolic enzymes involved in the model were highly conserved among the 31 *Streptomyces* strains. The strain S17-slv had highest homology (98%) and the stain S31-sxi had lowest homology (72%) with the all 1208 metabolic enzymes. Specially, 24 strains had higher homology more than 80%, and only 2 strains had homology least than 75% with the analyzed proteins in *S. coelicolor* A3(2). The conservation of metabolic enzymes from different metabolic subsystems in *Streptomyces* species was also investigated by cluster analysis. The conservation of different subsystems showed a similar distribution in all strains (Additional file 3: Figure S1). Interestingly, the metabolic diversity mainly stemmed from the subsystems related to secondary metabolism, including actinorhodin biosynthesis (01-ActB), calcium-dependent antibiotics biosynthesis (08-CDAB), germicidin biosynthesis (16-GerB) and undecylprodigiosin biosynthesis (42-UndB).

**Fig. 2:**
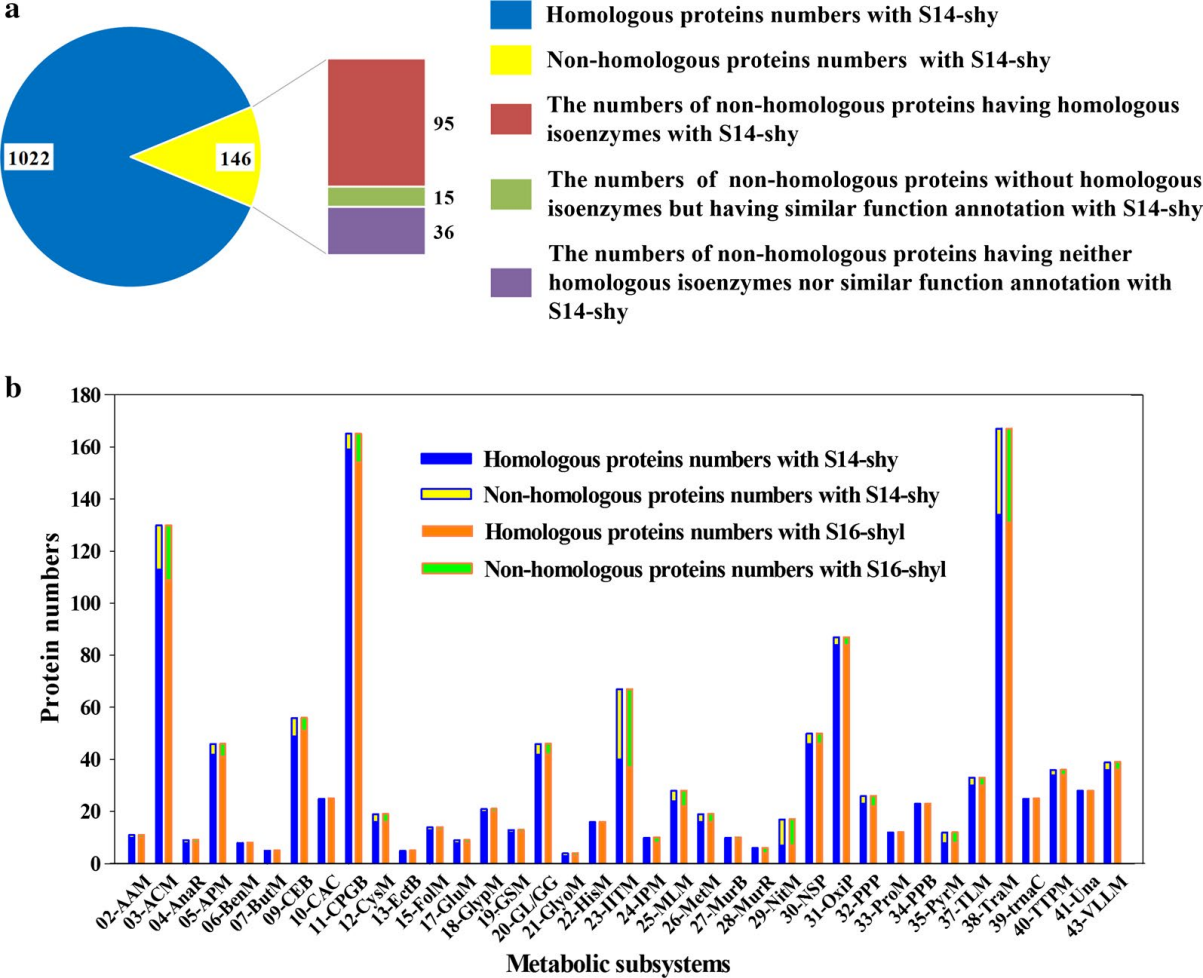
Conservation analysis of primary metabolic enzymes of *S. coelicolor* A3(2) compared with two *S. hygroscopicus* strains. The proteins involved in the primary metabolic subsystems of the metabolic model iMK1208 of *S. coelicolor* A3(2) were used as reference for protein conservation analysis of *S. hygroscopicus jinggangensis* 5008 (S14-shy) and *S. hygroscopicus limoneus* KCTC 1717 (S16-shyl). **a** Homology analysis of proteins related to primary metabolism in *S. coelicolor* A3(2) and S14-shy. The distribution of homologous and non-homologous proteins in the primary metabolic subsystems of the model iMK1208 against S14-shy is shown in the pie chart. The details of the numbers of non-homologous proteins owing isoenzymes or similar function annotation with the strain S14-shy are also listed in the bar of the pie chart. **b** Comparison of the proteins in different primary metabolic subsystems of the model iMK1208 with those from the strains S14-shy and S16-shyl respectively. Four secondary metabolism subsystems and two primary metabolism subsystems (not including any proteins in model) were not included in this analysis

In addition to the above significant differences in secondary metabolism, further concrete analysis of proteins within the primary metabolism was conducted using *S. hygroscopicus jinggangensis* 5008 (S14-shy) and *S. hygroscopicus limoneus* KCTC 1717 (S16-shyl) as examples. Homology analysis of 1166 primary metabolic enzymes from *S. coelicolor* A3(2) was preformed against S14-shy and S16-shyl. Both strains showed high numbers of enzymes homologous with those of the primary metabolic enzymes in *S. coelicolor* A3(2) (Fig. 2a and Additional file 3: Figure S2). In fact, only 146 and 169 proteins had no homologs in S14-shy and S16-shyl, respectively. It was also discovered that some of the non-homologous proteins might have homologous isoenzymes in the two strains. As for the strain S14-shy (Fig. 2a), it did not share significant sequence similarity with 146 proteins related to the primary metabolism of *S. coelicolor* A3(2). However, among these 146 proteins, there were 95 proteins with at least one isozyme present in S14-shy. Moreover, among the remaining 51 non-homologous proteins, 15 had similar protein annotation information with proteins in S14-shy. Taking all these factors into consideration, there were only 36 proteins without homologs among all 1166 primary metabolic related proteins from S14-shy, which represents a mere 3% of all proteins in the primary metabolism subsystems of the metabolic network model of *S. coelicolor* A3(2). Similar results were also obtained for the strain S16-shyl (Additional file 3: Figure S2). Moreover, the non-homologous proteins were mainly distributed in the subsystems related to environmental adaptability, such as alternate carbon metabolism (03-ACM), inorganic ion transport (23-IITM), nitrogen metabolism (29-NitM) and membrane transport (38-TraM) (Fig. 2b). Thus, there were only minor differences in the primary metabolism of the analyzed *Streptomyces* species.

### Metabolic network reconstruction and in silico identification of potential targets

According to the previous described methods (seen in the “Methods” section: Metabolic network construction), the metabolic network model of *S. hygroscopicus* var. *ascomyceticus* FS35 was constructed based on the model of *S. coelicolor* A3(2), and the details are listed in the Additional file 2. Using the maximum growth rate as an objective function, the constructed model was verified by comparing the specific growth rate from the simulation with experimental data. In the model simulation, the specific glucose uptake rate was set to 0.90 mM/g DCW/h and the specific ascomycin synthetic rate was set to 5 × 10^−4^ mM/g DCW/h respectively. Based on the above constraints, the maximal specific growth rate calculated by the model was 0.0653/h, which was very close to the experimental result of 0.0616/h (Additional file 3: Figure S3).

**Fig. 3:**
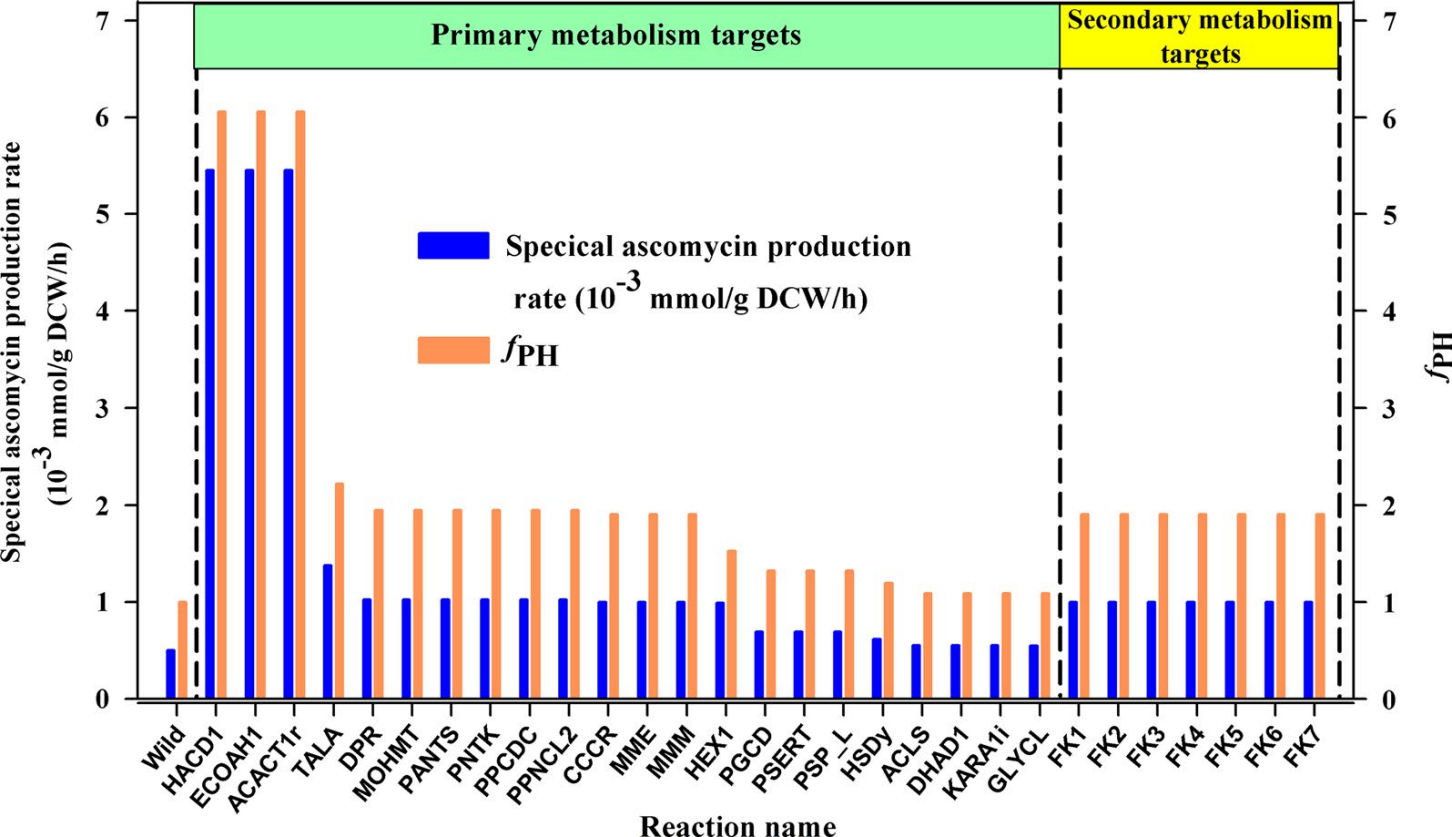
Predicted effects of single reaction perturbation on the specific ascomycin production and *f*_PH_ values

After model verification, the potential overexpression targets for ascomycin overproduction were predicted by coupling the model with FBA and MOMA algorithms. In order to improve the accuracy, targets located in the inorganic ion transport and metabolism, exchange and nucleotide salvage pathway subsystems were excluded in advance. The targets related to ATP maintenance requirements were also been excluded. Using their respective *f*_PH_ values [37], 29 potential gene overexpression targets were identified, including 22 from the primary metabolism and 7 from the secondary metabolism (Fig. 3). The details of all predicted targets are listed in Additional file 2. Among these, the targets HACD1, ECOAH1 and ACACT1r had the highest *f*_PH_ at 6.059. The corresponding reactions are catalyzed by 3-hydroxybutyryl-CoA dehydrogenase, 3-hydroxybutyryl-CoA dehydratase and acetyl-CoA C-acetyltransferase, respectively. All the three targets are involved in the transformation of acyl-CoA into crotonyl-CoA, and the latter is an important precursor for the supply of ethylmalonyl-CoA [48, 50]. The target TALA was catalyzed by transaldolase, which converts glyceraldehyde 3-phosphate and sedoheptulose 7-phosphate into d-erythrose 4-phosphate (E4P) and d-fructose 6-phosphate. In transaldolase-overexpressing strain, the carbon flux to E4P was enhanced which resulted in an increased supply of shikimate acid [38]. The targets MOHMT, PANTS, PNTK, PPNCL2, DPR and PPCDC are involved in the synthesis of pantetheine 4′-phosphate (an intermediate in coenzyme A biosynthesis pathway), which is an essential prosthetic group of acyl carrier protein (ACP), and a key cofactor in the biosynthesis of polyketides and fatty acids [51–53]. The next target, CCCR (*f*_PH_ = 1.906) was catalyzed by crotonyl-CoA carboxylase/reductase (Ccr). In this reaction, crotonyl-CoA could be transformed into ethylmalonyl-CoA, an important precursor in ascomycin biosynthesis [54]. Overexpressing the two targets MME and MMM, catalyzed by methylmalonyl-CoA epimerase and methylmalonyl-CoA mutase respectively, would also increase the precursor level of ascomycin biosynthesis. Additionally, the secondary metabolic reactions FK1, FK2, FK3, FK4, FK5, FK6 and FK7 were also predicted as overexpression targets, suggesting that engineering secondary metabolic pathways might improve the production of ascomycin. In fact, this strategy had been proven effective in promoting the production of many secondary metabolites production [55–58].

### Improving ascomycin production by engineering the ethylmalonyl-CoA pathway

**Fig. 4:**
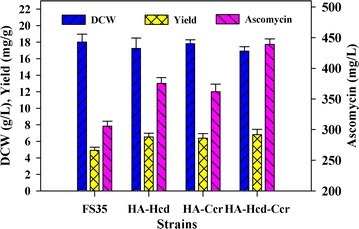
Experimental effects of target gene manipulation on ascomycin production and cell growth. The data represent the average values of at least three independent experiments and the error bars represent the standard deviations

**Fig. 5:**
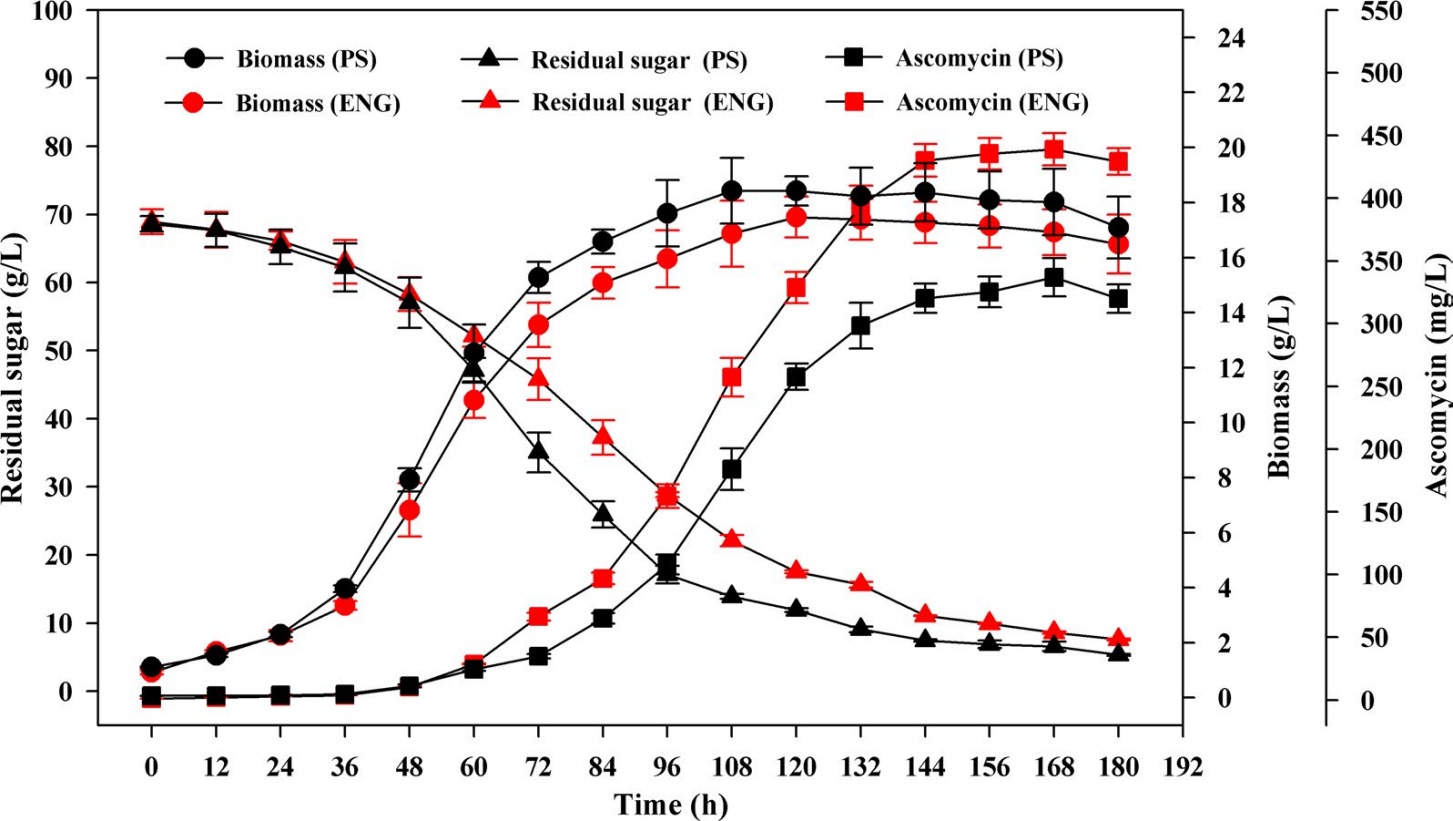
The fermentation kinetics of the parent strain *S. hygroscopicus* var. *ascomyceticus* FS35 and its derived engineered stain HA-Hcd-Ccr under the same culture conditions. PS indicates the parent strain FS35, and ENG indicates the engineered strain HA-Hcd-Ccr. The data represent the means of at least three series of three parallel tests, and the error bars represent standard deviations

According to the prediction results, it was found that several of the promising targets were highly associated with ethylmalonyl-CoA biosynthesis or directly located in the ethylmalonyl-CoA pathway. Consequently, two targets (HACD1 and CCCR) involved in the ethylmalonyl-CoA pathway were selected for the experimental overexpression. HACD1 and CCCR were catalyzed by 3-hydroxybutyryl-CoA dehydrogenase (Hcd) and crotonyl-CoA carboxylase/reductase (Ccr), respectively (Additional file 3: Table S2). To verify the accuracy of the model prediction, the *hcd* and *ccr* genes were individually overexpressed and co-overexpressed in *S. hygroscopicus* var. *ascomyceticus* respectively (Table 2). In the *hcd*-overexpressing strain HA-Hcd, the 3-hydroxybutyryl-CoA dehydrogenase activity was 0.49 U/mg protein, representing a 1.96-fold increase over the parent strain FS35 (0.25 U/mg protein). In the *ccr*-overexpressing strain HA-Ccr, the crotonyl-CoA carboxylase/reductase activity was 0.28 U/mg protein, an increase of 2.5 times over the control (0.08 U/mg protein). The enzymes activity in each mutant strain was higher than parental strain, which confirmed that both *hcd* and *ccr* genes were overexpressed. In addition, in the strain HA-Ccr, the 3-hydroxybutyryl-CoA dehydrogenase activity was 0.27 U/mg protein, a similar level with that of the control (0.25 U/mg protein). By contrast, in the *hcd*-overexpressed strain HA-Hcd, the crotonyl-CoA carboxylase/reductase activity was 0.13 U/mg protein, which was 1.62-fold higher than that of the control (0.08 U/mg protein). In the strain HA-Hcd, the ascomycin production reached up to 375.32 mg/L at 168 h, increased 1.23 times compared with that of the parent strain FS35 (305.56 mg/L) (Fig. 4). And in the strain HA-Ccr, the ascomycin production reached 361.67 mg/L at 168 h, representing 1.18-fold increase over the parent strain FS35 (Fig. 4). Importantly, there were no obvious changes in biomass concentration between the engineered strains HA-Hcd and HA-Ccr, and the parent strain FS35. Finally, the combined overexpression of *hcd* and *ccr* was also investigated. Similar with the overexpression of single gene, the growth of the double-overexpression strain HA-Hcd-Ccr was not significantly influenced. Its 3-hydroxybutyryl-CoA dehydrogenase activity was 0.55 U/mg protein, 1.2-fold higher than that of the control (0.25 U/mg protein). The crotonyl-CoA carboxylase/reductase activity was 0.35 U/mg protein, and thus was increased 3.37 times compared with that of the parent strain FS35 (0.08 U/mg protein). Enzyme activity analysis confirmed that the activity of 3-hydroxybutyryl-CoA dehydrogenase and crotonyl-CoA carboxylase/reductase activity in the strain HA-Hcd-Ccr was significantly improved compared with the control strain FS35. The final biomass concentration of the double-overexpression strain (16.92 g/L at 168 h) exhibited a slightly decrease compared with the parent strain FS35 (18.02 g/L at 168 h) (Fig. 5). Nevertheless, the final ascomycin production was obviously improved, reaching 438.95 mg/L at 168 h, which was a 1.43-fold increase over that of the parent strain FS35 (305.56 mg/L). The yield of ascomycin in HA-Hcd-Ccr was enhanced to 6.80 mg/g glucose from 4.91 mg/g glucose, and was thus 1.38-fold higher than that of the parent strain FS35.

**Table 2:** Specific activity of enzymes by parent strain *S. hygroscopicus* var. *ascomyceticus* FS35 and recombinants in batch cultures

Strains	Enzyme activites (U/mg protein)	
	Hcd	Ccr
FS35	0.25 ± 0.03	0.08 ± 0.01
HA-Hcd	0.49 ± 0.05	0.13 ± 0.01
HA-Ccr	0.27 ± 0.03	0.28 ± 0.03
HA-Hcd-Ccr	0.55 ± 0.05	0.35 ± 0.04

## Discussion

In previous reports, GSMM was confirmed as an effective method for better understanding of intracellular metabolic behavior and identifying the key limiting steps for target metabolites biosynthesis. In *Streptomyces* species, the GSMM of *S. coelicolor* A3(2) has been constructed and updated for 2 times and successfully used in the guide of metabolic engineering targets identity [18–20]. It has also been used as a model for guiding the constructing other metabolic network models for some *Streptomyces* species [13, 59]. However, though several GSMM had been constructed in some *Streptomyces* species, the conservation of these metabolic reactions existed in the reported GSMM had not been investigated completely. Previous studies showed that primary metabolism might be highly conserved between *Streptomyces* species [23, 24]. To investigate whether the primary metabolic reactions in the metabolic network model of *S. coelicolor* A3(2) are conserved in other *Streptomyces* species, a more comprehensive, deeper metabolic gene analysis of *Streptomyces* species was performed in this study.

The metabolic conservation of *Streptomyces* species was investigated by comparing the metabolic enzymes in the latest reported model of *S. coelicolor* A3(2) with those of 31 completely sequenced *Streptomyces* strains. The results showed that the metabolic enzymes were highly conserved within the 31 *Streptomyces* strains, especially for primary metabolism. This was consistent with the previous comparative genome and pangenome analyses of *Streptomyces* species [18, 23, 24]. Furthermore, only minor differences were founded in the primary metabolism of *Streptomyces* species, and metabolic reactions showed higher conservation than the data obtained through protein homology analysis. Therefore, majority of the primary metabolic reactions in the model of *S. coelicolor* A3(2) are likely universal across *Streptomyces* species. In addition, due to the conservation of metabolic genes and our limited knowledge of gene functions and biochemical characteristics, the set of primary metabolic reactions in *Streptomyces* strains is expected to converge to a constant value with the addition of analyzed genomes, as was demonstrated for *E. coli* strains [21, 22]. Considering the universal nature of reactions in the primary metabolism and the fact that strain-specific portions of the primary metabolism remain largely uncharacterized in *Streptomyces* species, it is reasonable to reference the primary metabolic reactions in the model of *S. coelicolor* A3(2) to construct general metabolic network models for other *Streptomyces* strains.

As an important application of the general metabolic model, the GSMM of *S. hygroscopicus* var. *ascomyceticus* was firstly attempted to be constructed and applied to identify the genetic engineering targets for improving ascomycin production. The constructed metabolic model of *S. hygroscopicus* var. *ascomyceticus* facilitated the understanding of the ascomycin biosynthesis network. Using this model in conjunction with the FBA and MOMA algorithms, we identified 29 potential gene overexpression targets according to a ranking of their respective *f*_PH_ values. Among all the predicted targets, several targets with high *f*_PH_ values (ACACT1r, HACD1, ECOAH1 and CCCR) were found in the ethylmalonyl-CoA pathway, which had also been confirmed to be an limiting pathway for other products synthesis in some *Streptomyces* species [54]. In fact, we also found that the ascomycin producing strain of *S. hygroscopicus* var. *ascomyceticus* FS35 could utilize acetate and ethylmalonic acid as a sole carbon source (data not shown), indicating the presence of the ethylmalonyl-CoA pathway in this strain. However, the ethylmalonyl-CoA pathway was incomplete in the most reported model of *Streptomyces* species. According to the conservative analysis of primary metabolism, the related metabolic reactions had been supplied into the general metabolic model by adding additional constraints and the missing reactions in the pathway (Additional file 4). Using the updated model, the targets related to the ethylmalonyl-CoA pathway were still predicted to have great relevance for ascomycin biosynthesis (Additional file 4). Therefore, the targets related to the ethylmalonyl-CoA pathway were successfully predicted to have great relevance for ascomycin biosynthesis (Additional file 4).

**Fig. 6:**
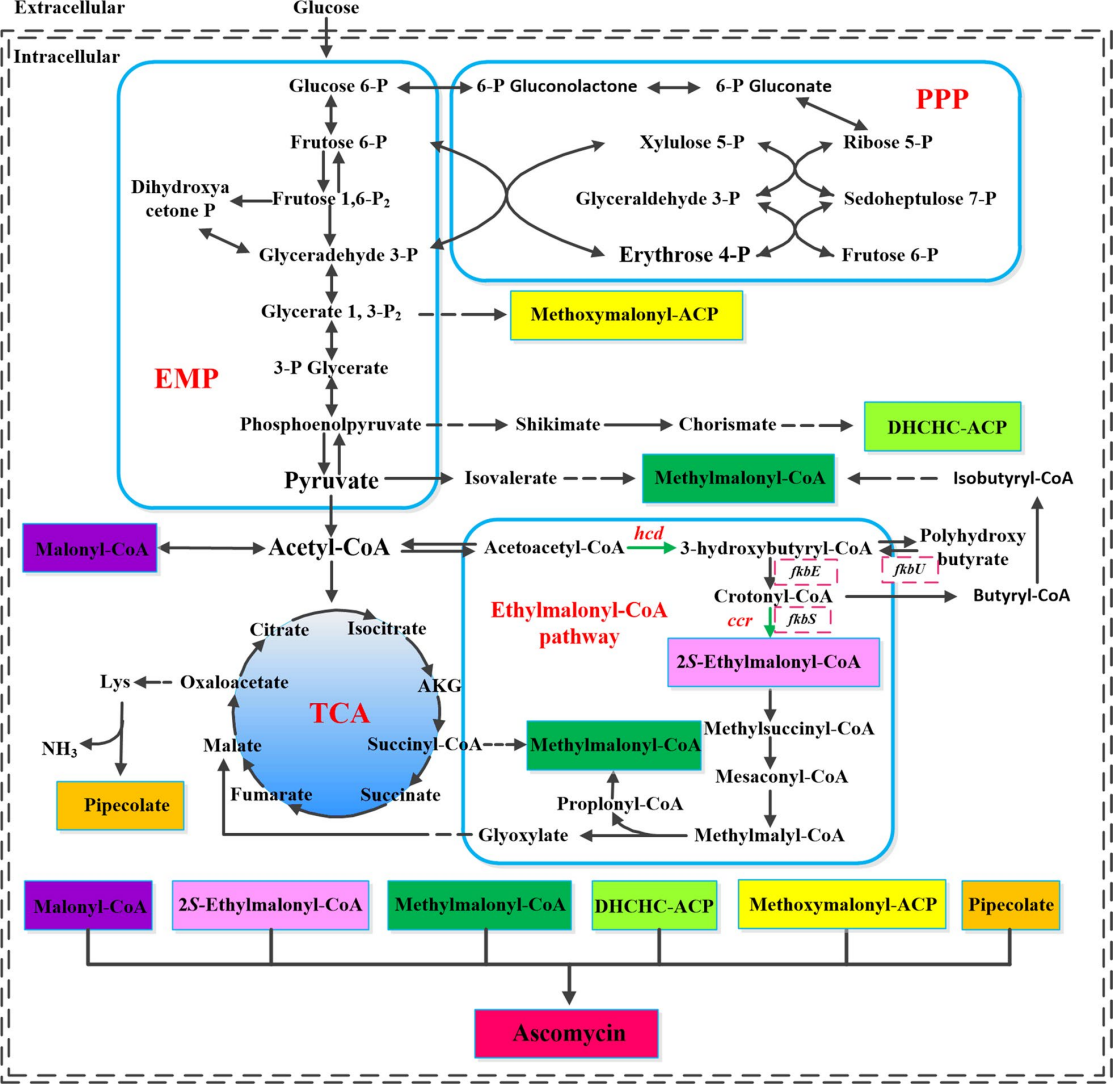
Schematic overview of the proposed ascomycin biosynthesis pathways in *S. hygroscopicus* var. *ascomyceticus*. Dotted lines indicate more than one reaction step and the shaded boxes represent precursors of ascomycin biosynthesis. The overexpression targets in this study for improved production of ascomycin are indicated with green arrows, and the genes included in the ascomycin biosynthesis gene cluster are shown in red broken line boxes

Among the above four targets (ACACT1r, HACD1, ECOAH1 and CCCR), HACD1 could converts acetoacetyl-CoA to 3-hydroxybutyryl-CoA through 3-hydroxybutyryl-CoA dehydrogenase encoded by *hcd*. Furthermore, it is worth mentioning that the metabolite 3-hydroxybutyryl-CoA plays important roles in the ethylmalonyl-CoA pathway and poly-hydroxybutyrate (PHB) biosynthesis (Fig. 6). PHB had been proved to be relevant to ascomycin biosynthesis in *S. hygroscopicus* var. *ascomyceticus* [60]. CCCR is another target involved in the ethylmalonyl-CoA pathway, corresponding to Ccr, which converts crotonyl-CoA to ethylmalonyl-CoA. This enzyme has been used to improve the production of several secondary metabolites in *Streptomyces* species, including monensin [61], tacrolimus [54], and salinomycin [58]. Furthermore, *hcd* and *ccr* are present in gene clusters encoding the ethylmalonyl-CoA pathway in all the examined *Streptomyces* strains (Additional file 3: Table S3). The target genes *hcd* and *ccr* were selected as the gene overexpression targets to further experimentally verify the accuracy of the model prediction and to enhance the production of ascomycin.

The two target genes *hcd* and *ccr* were overexpressed and co-overexpressed in *S. hygroscopicus* var. *ascomyceticus*. The experimental results suggested that all the engineered strains had a higher ascomycin production than control, and the highest ascomycin production could reach to 438.95 mg/L. The genetic manipulation about the target genes further verified the feasibility the strategy about model construction. However, the increase of production titer was different from the predicted improvements. This may be due to some limitations of the constraint-based metabolic network simulation, such as the lack of detailed knowledge on metabolic regulation, as well as thermodynamic and kinetic information. The strategy for metabolic model construction in *Streptomyces* species will make the construction process of metabolic network easier and make it possible for the strain without genomic information.

In addition, except for *S. hygroscopicus* var. *ascomyceticus*, the ethylmalonyl-CoA pathway is also found in many *Streptomyces* species, and the corresponding enzymes can be found in 30 of the 31 investigated *Streptomyces* strains (Additional file 3: Table S3). Enhancing the flux in ethylmalonyl-CoA pathway would improve the pools of many common precursor of in polyketide biosynthesis (Fig. 6), including ethylmalonyl-CoA, methylmalonyl-CoA, propionyl-CoA, crotonyl-CoA, butyryl-CoA, and so on [48]. In addition, the ethylmalonyl-CoA pathway overlaps with several important metabolic pathways, such as the PHB pathway, TCA cycle and serine cycle, and many PKS gene clusters associated with butyryl-containing metabolites also contain another copy of the *ccr* gene to supply ethylmalonyl-CoA [60, 62]. The complicated relationship between the ethylmalonyl-CoA pathway and other metabolic pathways might open new avenues for the improvement of polyketide production and rational pathway design.

## Conclusions

In this study, using the latest reported model of *S. coelicolor* A3(2) as model, a general metabolic model of *Streptomyces* species was attempted to be constructed based on the conservative analysis of the primary metabolic reactions of 31 *Streptomyces* strains. Using this general metabolic model, the GSMM of *S. hygroscopicus* var. *ascomyceticus* was reconstructed and applied to identify the potential targets for ascomycin overproduction. According to the simulated results, two targets *hcd* and *ccr* from the ethylmalonyl-CoA pathway were selected for overexpression, the experimental results indicated ascomycin production showed higher improvement in both of single- and double-overexpression strains. These findings could not only indicate that improving the supply of ethylmalonyl-CoA could improve ascomycin yield effectively, but also suggest that a general metabolic model could be existed and applied to guide for the yield improvement of other products in *Streptomyces* species.

## Additional files

**Additional file 1.** Conservation analysis of the proteins in the model iMK1208 against 31 *Streptomyces* strains.

**Additional file 2.** Metabolic network model of *S. hygroscopicus* var. *ascomyceticus* FS35 and potential targets identified using this model.

**Additional file 3: Table S1.** Primers used in this section. **Figure S1**. Heat map illustrating the conservation of metabolic enzymes in different metabolic subsystems among 31 *Streptomyces* strains. **Figure S2.** Homology analysis of proteins related to the primary metabolism of *S. coelicolor* A3(2) and S16-shyl. **Figure S3.** Production profiles of the stain *S. hygroscopicus* var. *ascomyceticus* FS35 in batch fermentation. **Table S2.** Genetic targets chosen for experimental implementation. **Table S3.** The encoded enzymes involved in the ethylmalonyl-CoA pathways of the 31 *Streptomyces* strains.

**Additional file 4.** The updated model including the complete ethylmalonyl-CoA pathway, and the potential targets identified using this model.
