# Supplementary material for: Metabolic network model guided engineering ethylmalonyl-CoA pathway to improve ascomycin production in Streptomyces hygroscopicus var. ascomyceticus
Source: Microb Cell Fact. 2017 Oct 3;16:169. doi: 10.1186/s12934-017-0787-5 (PMC5627430; doi:10.1186/s12934-017-0787-5)
Supplement: Supplementary file 3 — Additional file 3: Table S1. Primers used in this section. Figure S1. Heat map illustrating the conservation of metabolic enzymes in different metabolic subsystems among 31 Streptomyces strains. Figure S2. Homology analysis of proteins related to the primary metabolism of S. coelicolor A3(2) and S16-shyl. Figure S3. Production profiles of the stain S. hygroscopicus var. ascomyceticus FS35 in batch fermentation. Table S2. Genetic targets chosen for experimental implementation. Table S3. The encoded enzymes involved in the ethylmalonyl-CoA pathways of the 31 Streptomyces strains. [file 12934_2017_787_MOESM3_ESM.docx]

**Additional file 3**

**Table S1**

**Primers used in this section**

| Primer name | Sequence 5'→3' |
| --- | --- |
| hcdF1 | AATTACCATATGGCCACTCCCCTGTCCGACAC |
| hcdR1 | CTAGTCTAGATCCGCCCCAGTCCTCCTCG |
| hcdF2 | TTCAAGCTTATCCATATGATGGCCACTCCCCTGTCCGACAC |
| hcdR2 | AAAACTGCAGTCCGCCCCAGTCCTCCTCG |
| ccrF1 | GGAATTCCATATGACCGTGAAGGACATCCT |
| ccrR1 | CTAGTCTAGACCTTGTGACGCTCAGTCATGTG |
| ccrF2 | AATCTGCAGACCGTGAAGGACATCCT |
| ccrR2 | CTAGTCTAGATCAGATGTTCCGGAAGCGGT |
| pIB-F | CGATGCTGTTGTGGGCACA |
| pIB-R | CGCGTTGGCCGATTCAT |

**Figure S1**


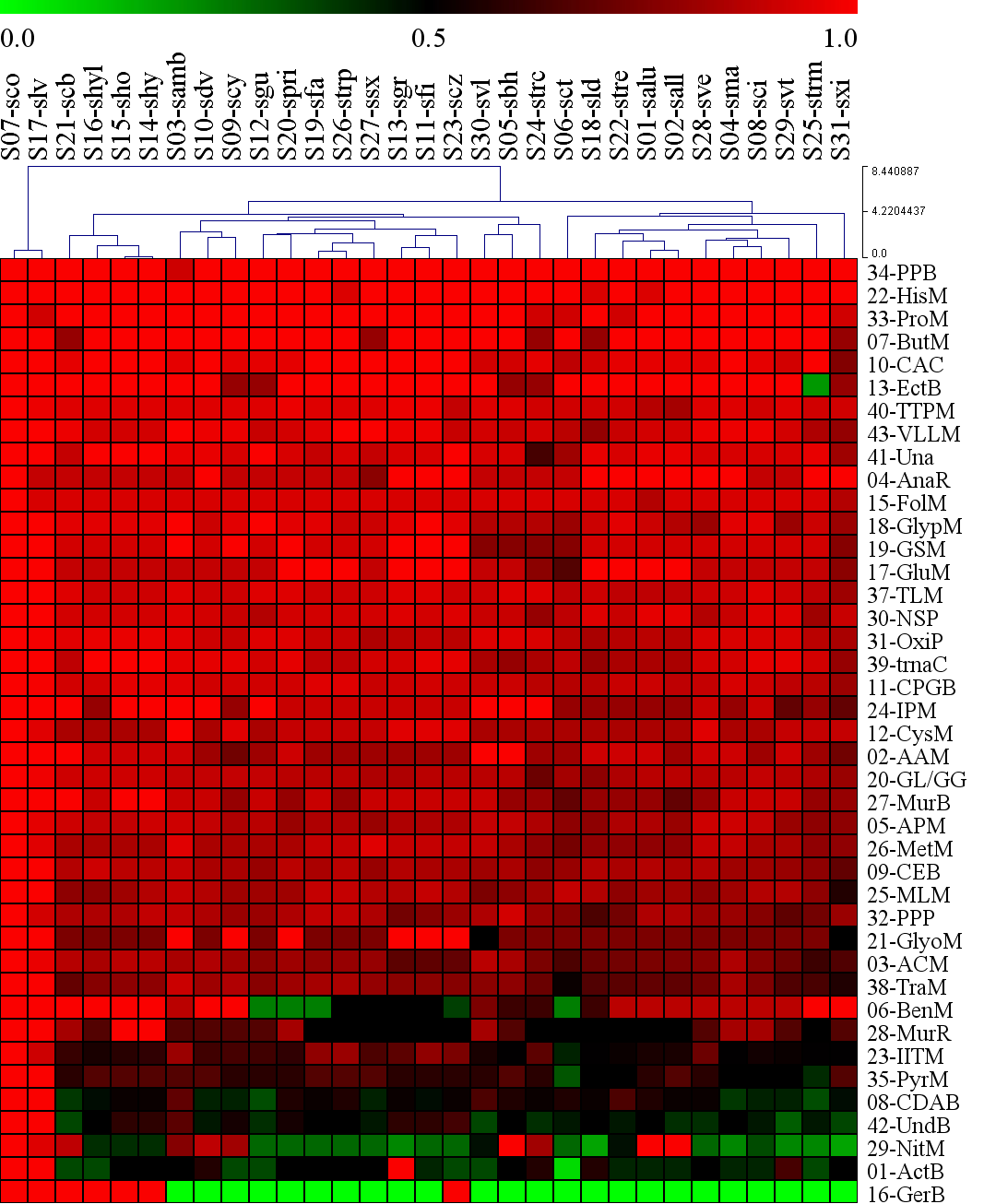


**Figure S1**

Heat map illustrating the conservation of metabolic enzymes in different metabolic subsystems among 31 *Streptomyces* strains. Metabolic subsystems are graphed in the order top to bottom according the average homology of metabolic enzymes in different metabolic subsystems of *S. coelicolor* A3(2) compared with 31 *Streptomyces* strains. Exchange and Teichoic Acid Biosynthesis of metabolic subsystems are not included in the analysis for not involving any enzymes in the model iMK1208. 01-ActB, Actinorhodin Biosynthesis; 02-AAM, Alanine and Aspartate Metabolism; 03-ACM, Alternate Carbon Metabolism; 04-AnaR, Anaplerotic Reactions; 05-APM, Arginine and Proline Metabolism; 06-BenM, Benzoate Metabolism; 07-ButM, Butanoate Metabolism; 08-CDAB, Calcium-Dependent Antibiotics Biosynthesis; 09-CEB, Cell Envelope Biosynthesis; 10-CAC, Citric Acid Cycle; 11-CPGB, Cofactor and Prosthetic Group Biosynthesis; 12-CysM, Cysteine Metabolism; 13-EctB, Ectoine Biosynthesis; 15-FolM, Folate Metabolism; 16-GerB, Germicidin Biosynthesis; 17-GluM, Glutamate Metabolism; 18-GlypM, Glycerophospholipid Metabolism; 19-GSM, Glycine and Serine Metabolism; 20-GL/GG, Glycolysis/Gluconeogenesis; 21-GlyoM, Glyoxylate Metabolism; 22-HisM, Histidine Metabolism; 23-IITM, Inorganic Ion Transport and Metabolism; 24-IPM, Inositol Phosphate Metabolism; 25-MLM, Membrane Lipid Metabolism; 26-MetM, Methionine Metabolism; 27-MurB, Murein Biosynthesis; 28-MurR, Murein Recycling; 29-NitM, Nitrogen Metabolism; 30-NSP, Nucleotide Salvage Pathway; 31-OxiP, Oxidative Phosphorylation; 32-PPP, Pentose Phosphate Pathway; 33-ProM, Propanoate Metabolism; 34-PPB, Purine and Pyrimidine Biosynthesis; 35-PyrM, Pyruvate Metabolism; 37-TLM, Threonine and Lysine Metabolism; 38-TraM, Transport, Membrane; 39-trnaC, tRNA Charging; 40-TTPM, Tyrosine, Tryptophan, and Phenylalanine Metabolism; 41-Una, Unassigned; 42-UndB, Undecylprodigiosin Biosynthesis; 43-VLLM, Valine, Leucine, and Isoleucine Metabolism

**Figure S2**

**Figure S2**

Homology analysis of proteins related to the primary metabolism of *S. coelicolor* A3(2) and S16-shyl. The homologous and non-homologous proteins in the primary metabolism subsystems of the model iMK1208 against S16-shyl are counted and list in the pie chart. Among the non-homologous proteins, many of them have isozymes, and some of which were homologous with the proteins in the strains, in addition, many non-homologous proteins had similar annotation information with the proteins of the analyzed strains. The details of the numbers of non-homologous proteins owing isoenzymes and similar function annotation with the proteins in the strain are listed in the bar of pie chart.

**Figure S3**

**Figure S3**

Production profiles of the stain *S. hygroscopicus* var. *ascomyceticus* FS35 in batch fermentation. Fermentation characteristics are detected using improved ISP4 culture medium. The data are the means of at least three series of three parallel tests, and the error bars represent standard deviations.

**Table S2**

Genetic targets chosen for experimental implementation

| **Gene** | **ORF** | **EC** | **Reaction stoichiometry** | **enzyme** |
| --- | --- | --- | --- | --- |
| *hcd* | SCO6475 | 1.1.1.157 | (S)-3-Hydroxybutanoyl-CoA + NADP+ <=> Acetoacetyl-CoA + NADPH + H+ | 3-hydroxybutyryl-CoA dehydrogenase |
| *ccr* | SCO6473 | 1.3.1.86 | Butanoyl-CoA + NADP+ <=> Crotonoyl-CoA + NADPH + H+ | crotonyl-CoA carboxylase/reductase |

ORF: open reading frame, *EC:* enzyme commission number

**Table S3**

The encoded enzymes involved in the ethylmalonyl-CoA pathways of the 31 *Streptomyces* strains

| Strain ID | Strain Name | Hcd | Ccr | MeaA | Msd |
| --- | --- | --- | --- | --- | --- |
| S01-salu | *S. albulus* NK660 | AIA06924.1 | AIA06922.1 | AIA06921.1 | AIA06918.1 |
| S02-sall | *S. albulus* ZPM | AKA06776.1 | AKA06774.1 | AKA05589.1 | AKA06770.1 |
| S03-samb | *S. ambofaciens* ATCC 23877 | AKZ59115.1 | AKZ59112.1 | AKZ59111.1 | AKZ59108.1 |
| S04-sma | *S. avermitilis* MA-4680 | BAC69620.1 | BAC69622.1 | BAC69623.1 | BAC69626.1 |
| S05-sbh | *S. bingchenggensis* BCW-1 | ADI05953.1 | ADI05957.1 | ADI05958.1 | ADI05960.1 |
| S06-sct | *S. cattleya* NRRL 8057 | AEW97226.1 | AEW97224.1 | AEW97223.1 | AEW97221.1 |
| S07-sco | *S. coelicolor* A3(2) | CAA22723.1 | CAA22721.1 | CAA22720.1 | CAA22717.1 |
| S08-sci | *S. collinus* Tu 365 | AGS72762.1 | AGS72760.1 | AGS72759.1 | AGS72756.1 |
| S09-scy | *S. cyaneogriseus noncyanogenus* NMWT 1 | AJP04640.1 | AJP04639.1 | AJP04638.1 | AJP04636.1 |
| S10-sdv | *S. davawensis* JCM 4913 | CCK26329.1 | CCK26331.1 | CCK26332.1 | CCK26335.1 |
| S11-sfi | *S. fulvissimus* DSM 40593 | AGK81214.1 | AGK81210.1 | AGK81209.1 | AGK81206.1 |
| S12-sgu | *S. glaucescens* GLA.O | AIS01391.1 | AIS01389.1 | AIS01388.1 | AIS01385.1 |
| S13-sgr | *S. griseus subsp. griseus* NBRC 13350 | BAG17995.1 | BAG17999.1 | BAG18000.1 | BAG18003.1 |
| S14-shy | *S. hygroscopicus jinggangensis* 5008 | AEY92703.1 | AEY92701.1 | AEY92700.1 | AEY92697.1 |
| S15-sho | *S. hygroscopicus jinggangensis* TL01 | AGF66858.1 | AGF66856.1 | AGF66855.1 | AGF66852.1 |
| S16-shyl | *S. hygroscopicus limoneus* KCTC 1717 | ALO97351.1 | ALO97349.1 | ALO97348.1 | ALO97345.1 |
| S17-slv | *S. lividans* TK24 | AIJ12225.1 | AIJ12227.1 | AIJ12228.1 | AIJ12231.1 |
| S18-sld | *S. lydicus* A02 | AJT63399.1 | AJT63402.1 | AJT63403.1 | AJT63406.1 |
| S19-sfa | *S. pratensis* ATCC 33331 | ADW02359.1 | ADW02363.1 | ADW02364.1 | ADW02367.1 |
| S20-spri | *S. pristinaespiralis* HCCB 10218 | ALC19634.1 | ALC19636.1 | ALC19637.1 | ALC19640.1 |
| S21-scb | *S. scabiei* 87.22 | CBG68890.1 | CBG75361.1 | CBG68893.1 | CBG68896.1 |
| S22-stre | *S. sp.* 769 | AJC54912.1 | AJC54916.1 | AJC54917.1 | AJC54920.1 |
| S23-scz | *S. sp.* CFMR 7 | ALC26451.1 | ALC26455.1 | ALC26456.1 | ALC26459.1 |
| S24-strc | *S. sp.* CNQ-509 | AKH85555.1 | AKH85553.1 | AKH85552.1 | AKH85549.1 |
| S25-strm | *S. sp.* Mg1 | AKL68573.1 | AKL68571.1 | AKL68570.1 | AKL68567.1 |
| S26-strp | *S. sp.* PAMC26508 | AGJ58381.1 | AGJ58377.1 | AGJ58376.1 | AGJ58373.1 |
| S27-ssx | *S. sp.* SirexAA-E | AEN13454.1 | AEN13450.1 | AEN13449.1 | AEN13446.1 |
| S28-sve | *S. venezuelae* ATCC 15439 | ALO11957.1 | ALO11955.1 | ALO11954.1 | ALO11951.1 |
| S29-svt | *S. vietnamensis* GIM4.0001 | AJF68095.1 | AJF68093.1 | AJF68092.1 | AJF68089.1 |
| S30-svl | *S. violaceusniger* Tu 4113 | AEM82225.1 | AEM82221.1 | AEM82220.1 | AEM82217.1 |
| S31-sxi | *S. xiamenensis* 318 | - | - | - | - |

Hcd: 3-hydroxyacyl-CoA dehydrogenase; Ccr: crotonyl-CoA carboxylase/reductase; MeaA: methylmalonyl-CoA mutase; Msd: methylsuccinyl-CoA dehydrogenase; -, no examined.
